# Evaluating the Non-Invasive Measurement of Apple Aroma Using Electronic Nose Device through Comparison with Direct Mass Spectrometry, Sugar Content, and Ripeness Measurements

**DOI:** 10.3390/s24103114

**Published:** 2024-05-14

**Authors:** Kouki Fujioka

**Affiliations:** Research Center for Agricultural Information Technology, National Agriculture and Food Research Organization (NARO), 2-14-1 Nishi-Shimbashi, Minato-ku, Tokyo 105-0003, Japan; fujioka_koki@affrc.go.jp; Tel.: +81-3-3503-6660

**Keywords:** e-nose, direct mass spectrometry, apple, aroma

## Abstract

To compare apple aroma intensities, apples were analyzed from the calyx side (on the opposite side of the stem) using an electronic nose (e-nose) sensor device and direct mass spectrometry. The results indicated that the sensor value tended to increase in accordance with the total intensity of apple aroma components measured by direct mass spectrometry. In addition, the e-nose sensor values for apple aroma did not correlate with the sugar content and ripeness measurements using optical sensors. Moreover, the relative standard deviations of repeatability and intermediate precision in the measurement of apple flavor (apple lip balm) were within 1.36–9.96%. Similar to the utilization of sugar content and ripeness values, the aroma measured from the calyx side can be potentially used for apple evaluation.

## 1. Introduction

As a technology to support human sensory evaluation and quality evaluation of food items, research on the development and utilization of the electronic nose (e-nose) are ongoing. For example, e-noses can be used to evaluate the quality of food items such as milk and dairy products, wine, tea, coffee, meat, fish, chocolate, alcoholic beverages, and fruits [1,2].

Apples are popular because of their nutrients and bioactive compounds [3]. As their aroma is indicative of the quality and ripeness, e-nose systems were developed to investigate apple aroma in previous studies [4]. For example, Zou et al. [5] reported a novel e-nose grading system for apple quality based on computational fluid dynamics simulations; and Saevels et al. [6] reported an apple quality assessment system during shelf life. In addition, e-noses have been used to predict the optimal harvest date or ripeness of apples [7], as well as to develop a detection system for moldy apples [8].

The aroma of apples varies with variety and is one of the factors that determines palatability [9,10]. Moreover, apples contain approximately 300 aroma components [11]. In Fuji apples, esters such as ethyl 2-methylbutyrate and methyl 2-methylbutyrate are the main contributors to flavor [12]. In a study, by using Fuji apple fragrances with different ester concentrations, correlations were observed between the concentration of ethyl esters (ethyl hexanoate as a representative) and the scores of overall aroma intensity and sweetness in sensory evaluations [13]. Aroma is thus an important factor affecting the palatability and flavor of apples. 

Aromatic components are typically measured using gas chromatography-mass spectrometry (GC-MS), which can detect numerous components. However, this may not accurately reflect the actual composition of the released aroma components when using extraction or carriers to adsorb volatile organic compounds (VOCs). Therefore, the GC-MS using such methods and the e-nose devices that measure aroma components directly may give different results. To address this issue, we focused on a direct mass spectrometry system. The direct mass spectrometry system directly ionizes and measures VOCs emitted in the air at an ambient pressure [14], resulting in a composition of the measured components that was considerably closer to that of the actual emitted components. 

In our previous study [15], we developed a novel e-nose device to measure a sample cheese aroma by simply placing it on the device. In the study, although the intensities of the cheese aroma were measured from the bottom side [15], preliminary results indicated that the e-nose device could also measure the aroma of apples also from the calyx side by simply placing it on the same device [16]. By establishing a correlation between the e-nose sensor values and the aroma components, this method can be potentially used for predicting sensory evaluation scores. 

This study compared the values obtained from an e-nose sensor device with those obtained from direct mass spectrometry to verify the accuracy of measuring apple aroma from the calyx side. The reproducibility and intermediate precision (inter-day errors) of aroma measurements from the bottom side were also tested using an apple-flavored lip balm. Furthermore, the e-nose sensor values were compared with the sugar content and ripeness values obtained using optical sensors, which are currently used to assess apple quality.

## 2. Materials and Methods

### 2.1. Specifications of the E-Nose Device and Aroma Measurement Procedure

The use of an e-nose device has been described in detail in our previous study [15]. The device consists of a circular top plate, housing, and one metal oxide semiconductor sensor. A hole in the top plate enables the measurement of aroma. A pump inside the housing aspirates the aroma components below a sample through the hole, and the semiconductor sensors in the housing detects the aroma components. When the sample measurement was initiated, the e-nose device first exhibited an increase in the measurement value, followed by a stable or peak value. In this study, the sensor values were considered as peak or stable values. Prior to aroma measurement, the e-nose device stabilized the sensor value by measuring the air in the room for approximately 10 min.

**Figure 1:**
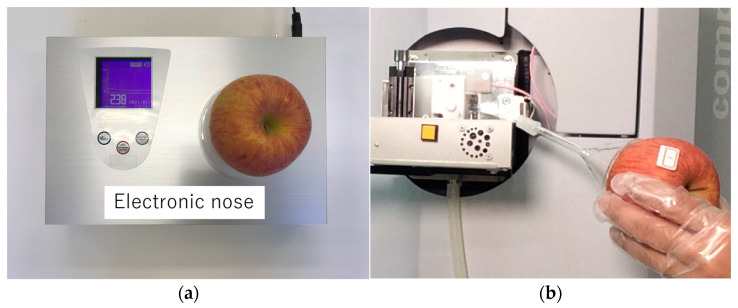
Measurement of apple aroma from the calyx side: (**a**) with the e-nose device; (**b**) with direct mass spectrometry.

This study used Sun Fuji apples sold at several retail stores in Japan as samples. The mean value and standard deviation were calculated by measuring three times from the calyx side, which is on the opposite side to the apple’s top side (stem side), at room temperature (23–24 °C) (Figure 1a). The relative standard deviation (RSD%) was determined by dividing the standard deviation of three sensor values by the mean.

Samples that underwent mass spectrometry analysis of the volatile aroma components differed from those that underwent sugar content and ripeness measurements. The sugar content and ripeness of 20 samples, 10 in February and 10 in March 2021, were measured over 5 days to ensure the robustness of the measurements.

### 2.2. Direct Mass Spectrometry Analysis

To investigate the correlation between the signal intensity of mass spectrometry and the sensor values, three apples with sensor averages differing by more than 30 were selected. Mass spectrometry was performed the day after the e-nose measurements. To conduct nondestructive analysis in the same manner as the e-nose sensor device, volatile components were aspirated from the calyx side using a glass funnel (diameter: 6 cm) at room temperature (23 °C) (Figure 1b). For the ionization of the components, a multipurpose ion source, ChemZo device (BioChromato, Kanagawa, Japan), which can ionize by corona discharge [14], was used. In addition, the molecular weight (*m*/*z*: 30–1000, positive ion mode) was measured using a compact QTOF instrument (Bruker, Billerica, MA, USA). In this study, the observed molecular weights were assumed to be ester-like molecules (C_4_H_8_O_2_~C_12_H_24_O_2_). Measurements were performed in triplicate for each sample to calculate the mean and standard deviation. The measurements of volatile components using the direct mass spectrometry were performed under commission by BioChromato.

### 2.3. Repeatability and Intermediate Precision of the E-Nose Device

**Figure 2:**
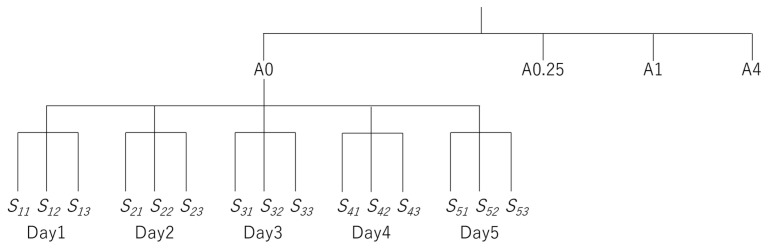
Nested design image for the five measurement days in this study. A0–A4 indicate balm-coated area (0–4 cm^2^). S_xy_ indicates sensor value for measurement number y on day x.

An apple-scented lip balm (Delicious Lip Cream, SUNSMILE Inc., Tokyo, Japan) was used as a standard to measure the scent of the apples. Lip balms are advantageous because they can be easily applied without the need for special tools. As a method of coating, a square (0, 0.25, 1, or 4 cm^2^) was drawn on a piece of paper (7.5 cm × 7.5 cm) and lip balm was applied inside the square. The paper was immediately aligned with the center of the hole on the top plate of the e-nose device and the sensor measured the VOCs from the back of the paper. The paper itself (not lip balm-coated) was used as a control (0 cm^2^). These measurements and applications of the balm were performed in triplicate per area. These measurements were repeated on five different days (four levels of concentration (coated area) × three measurements × five days; Figure 2), and the average value and standard deviation were calculated. The approximate curve formula was calculated using the MATLAB curve-fitting toolbox (MathWorks, Natick, MA, USA).

Repeatability and intermediate precision were calculated using Equations (1)–(5), referring to the calculation method described in the “Guideline for Validation of Test Methods for Pesticide Residues in Food (in Japanese)” [17]. In this study, the e-nose value measured in each balm-coated area was used as an alternative method to assess the repeatability and intermediate precision of the component concentration. One-way analysis of variance (ANOVA) was performed using the “Data Analysis” tools in Excel (Microsoft, Redmond, WA, USA) to calculate the repeatability (*σ_r_*) from the mean square (*Ms*) of the parallel studies (“*Within Group*”) (1). Using *N* (the number of tests per day), *σ_r_*, and the *Ms* of “*Between Groups*”, the standard deviation of the population mean of each day (*σ_d_*) was calculated (4). The intermediate precision was then calculated using *σ_r_* and *σ_d_* (5). Referring to the calculations in the guidelines [17], the respective relative standard deviations (RSD%) were determined by dividing by the mean of the e-nose sensor values in each applied area.

(1) $$\mathrm{Repeatability}=\sigma_{r}=\sqrt{Ms\;(“Within\;Group”)}$$

(2) $$Standard\;Deviation\;of\;the\;population\;mean\;of\;each\;day=\sigma_{d}$$

(3) The number of tests per day=N

(4) $$Ms(“Between\;Groups”)={\sigma_{r}}^{2}+{{N\sigma }_{d}}^{2}$$

(5) $$Intermediate\;precision=\sqrt{{\sigma_{r}}^{2}+{\sigma_{d}}^{2}}$$

### 2.4. Determination of Sugar Contents and Ripeness

The sugar content (Brix) and ripeness of apples were measured on the same day as the e-nose measurement. A nondestructive brix meter PAL-HIKARi 5 (Atago, Tokyo, Japan) was used to measure sugar content (%), and a nondestructive ripeness meter PAL-HIKARi Ripeness (Atago) was used to measure ripeness (%). According to the manufacturer’s instructions, the side surface of the fruit was mounted on the sensor and measured three times by changing the surface position to be measured, and the average values and sample standard deviations were calculated.

### 2.5. Statistical Analysis

Statistical analyses were performed using IBM SPSS Statistics Version 28 (IBM, Armonk, NY, USA) or Excel (Microsoft) with a significance level of 5%. A one-way ANOVA was performed to compare the e-nose sensor values, intensity values of mass spectrometry, sugar content, and ripeness. Tukey’s honestly significant difference (Tukey’s HSD) test was used to compare the groups. The relative standard variation (RSD%) was determined by dividing the sample standard deviation by the mean of the sensor values measured in triplicate for each apple, and the coefficients of variation for the 20 samples were compared. In addition, Pearson correlation coefficients and decision tree were calculated using the IBM SPSS Statistics.

## 3. Results and Discussion

### 3.1. Comparison of Mass Spectrometry Intensity and E-Nose Values for Apple Aroma

**Figure 3:**
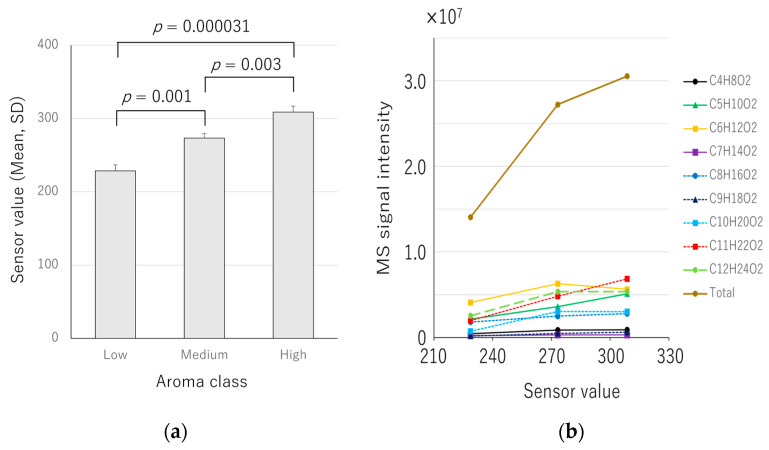
Comparison of e-nose sensor values and the intensities of direct mass spectrometry. (**a**) E-nose sensor value of each aroma class; and (**b**) signal intensities of each ester-like molecule and the e-nose sensor values.

The apple samples were placed on the top plate of the e-nose device and measured from the calyx side (Figure 1a). Three apples with sensor averages differing by more than 30 were selected to investigate the correlation between the sensor values and the signal intensity of mass spectrometry. The three apples were tested, and they exhibited different sensor values: 228.7 ± 8.14 (low), 273.3 ± 6.11 (medium), and 308.7 ± 8.14 (high), with a difference of more than 30 sensor values. (Figure 3a). These sensor values showed significant differences in a one-way ANOVA (*p* = 0.000039); in addition, significant differences were observed between low and medium (*p* = 0.001), low and high (*p* = 0.000031), and medium and high (*p* = 0.003) (Figure 3a).

**Figure 4:**
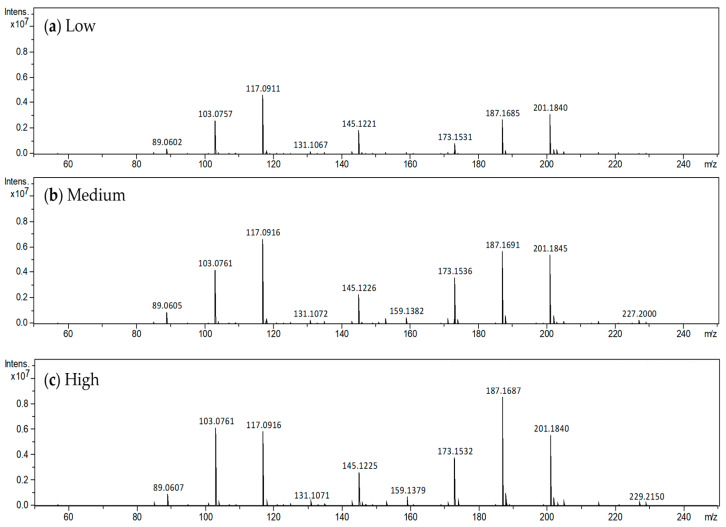
Representative data from direct mass spectrometry of apples displaying (**a**) low, (**b**) medium, and (**c**) high sensor value.

**Figure 5:**
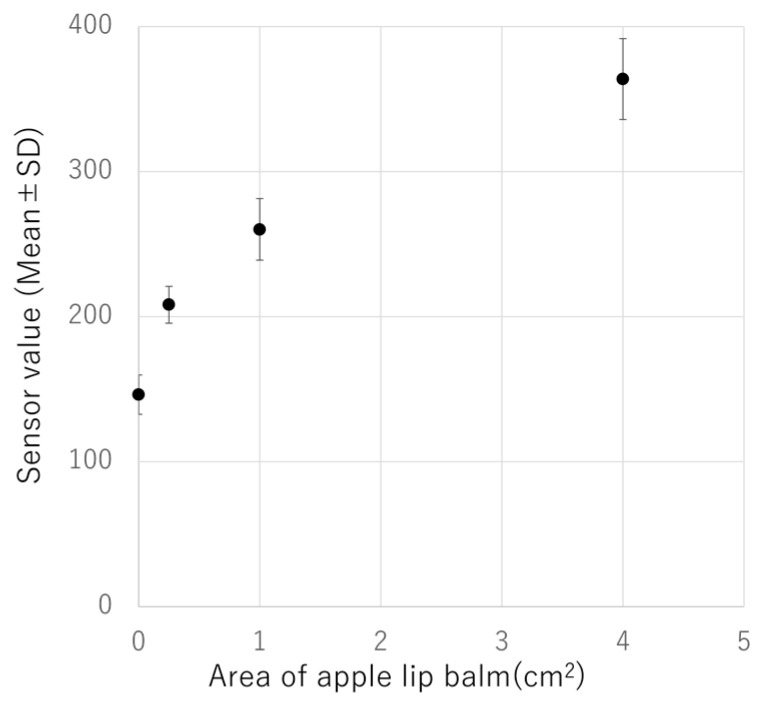
E-nose sensor values and coating area of apple lip balm (measurements over five days). Approximate curve: (sensor value) = 121.0 ∗ (area of apple lip balm) ^0.440^ + 140.8.

In order to confirm the results of the e-nose sensor, the volatile components of the apple were aspirated from the calyx side in a nondestructive manner and analyzed using a direct mass spectrometer system with corona discharge ionization [14]. From the calyx side, molecular weights ranging from 89.06 to 201.18 were mainly detected. In the previous study about the VOC of Fuij apples [18], ester and alcohol components were responsible for almost all of the total chromatographic area in GC-MS measurements, 89.9% and 6.0%, respectively. Therefore, the molecular weights detected in this study were assumed to be that of the ester-like molecules mainly, C_4_H_8_O_2_–C_12_H_24_O_2_ (Figure 4, Table 1). The total MS signal intensity values of these components were highest in the sample with high e-nose value, followed by the medium and low e-nose values (Table 1, Figure 3b). Therefore, the sensor value tended to be higher when the MS signal intensities of the ester-like components were higher. Furthermore, there was a correlation between the sensor values and the area of the lip balm containing apple flavor (Figure 5), suggesting that the e-nose device exhibited a value according to the amount of apple aroma components. Six of the nine intensities of MS signals (C_4_H_8_O_2_, C_5_H_10_O_2_, C_7_H_14_O_2_, C_8_H_16_O_2_, C_9_H_18_O_2_, and C_11_H_22_O_2_) had the same order of magnitude as the sensor values (Table 1).

### 3.2. Repetability and Intermediate Precision of the E-Nose Measurement

When measuring samples over multiple days, it is necessary to ensure the stability of the sensor values. To verify the stability of the measurements with the e-nose device for bottom side aroma, we assessed its repeatability and intermediate precision using an apple-flavored lip balm. The sensor values tended to increase with an increase in the coating area. Accuracy verification using flavor, including control results, showed a repeatability of 1.36–7.81% and indoor precision of 6.23–9.96% for four concentrations (coated area) × three measurements each for five days, both of which were found to have less than 10% error (Table 2). 

Focusing on the measurement accuracy of other measurement methods for comparison, although different from the sensor measurement, there is a report on the validation study on the headspace-GC analytical method for residual volatile components in food contact polystyrene and its application [19]. In the validation study, targets with a repeatability of <10% and intermediate precision of <15% were adopted. Their repeatability ranged from 3.7 to 6.3%, and the intermediate precision ranged from 6.0 to 11.1%. Compared to these accuracies, the e-nose device used in this study for apple aroma measurement was found to have a similar intermediate precision; however, its repeatability error was slightly greater than that of the headspace-gas chromatography (GC) measurement reported in this study. Headspace-GC is a reliable technique that is widely used to measure volatile components. Although a direct comparison is difficult owing to differences in the detected components and concentrations, the e-nose sensor value measured by this device had an error that is less than 10%, which was the target value in the previously reported headspace-GC method. This finding suggests that the e-nose measurement has a certain degree of stability.

### 3.3. Comparison of the Value of E-Nose Sensor with Sugar Contents or Ripeness

**Figure 6:**
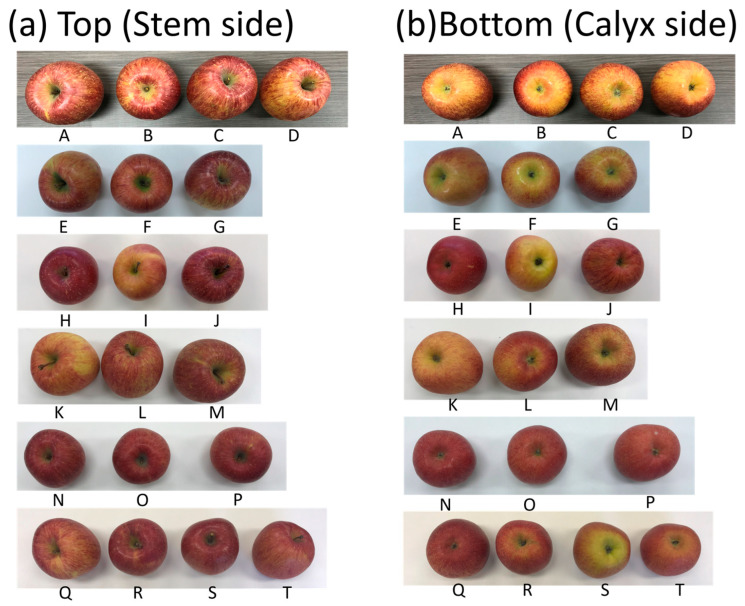
Apple samples (A–T) for the comparison of e-nose sensor (aroma), sugar content, and ripeness values. (**a**) Top (stem side) image, and (**b**) bottom (calyx side) image.

**Figure 7:**
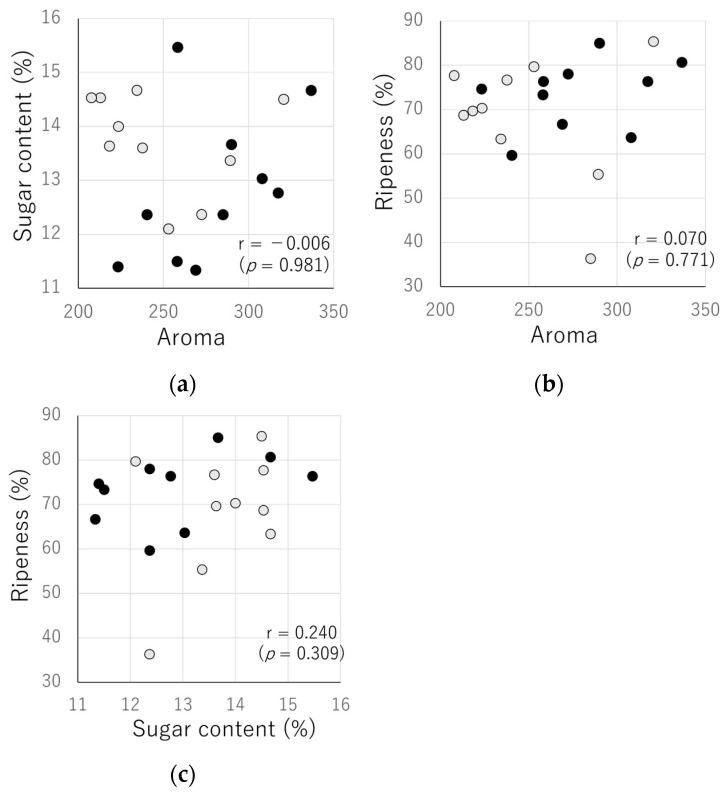
Comparison of e-nose sensor values (aroma), sugar content, and ripeness. (**a**) aroma and sugar content; (**b**) aroma and ripeness; (**c**) sugar content and ripeness (●: February, ●: March).

Objective indices, such as sugar content, ripeness, acidity, and hardness, are generally used to evaluate the quality of apples [20]. Therefore, we investigated the correlation between the sugar content and ripeness, which can be measured non-destructively, and the aroma intensity using the e-nose device. Therefore, the aroma intensity, sugar content, and ripeness were measure for 20 apple samples (Figure 6, Table 3). The correlations between aroma intensity and sugar content, aroma intensity and ripeness, and sugar content and ripeness were −0.006 (*p* = 0.981), 0.070 (*p* = 0.771), and 0.240 (*p* = 0.309), respectively (Figure 7). No significant correlations were observed between the measurement results. 

**Figure 8:**
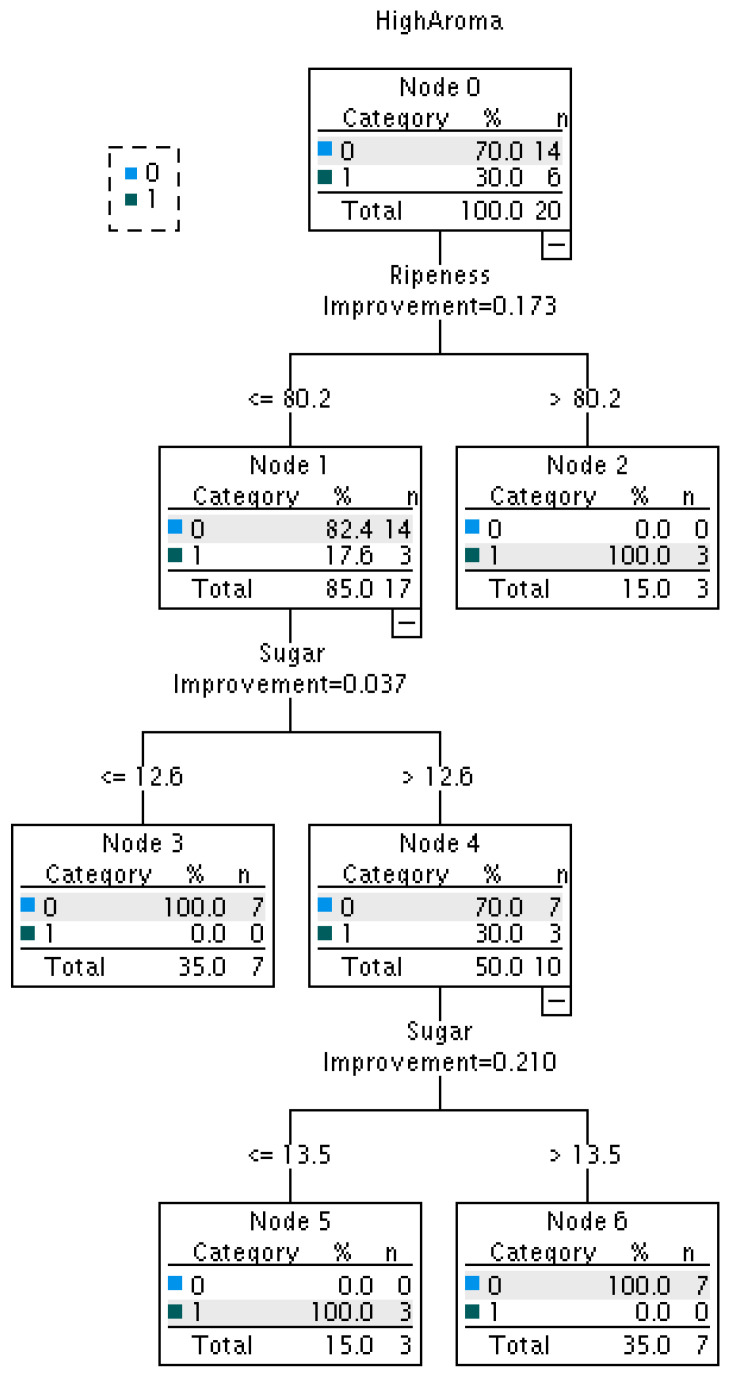
Decision tree for the top 30% of high aroma intensity apples (■1, aroma intensity > 289) using sugar content and ripeness values of 20 apples. Decision tree was calculated using IBM SPSS Statistics 28. ■0 indicates aroma intensity ≤ 289.

The decision tree analysis revealed that “apples with high ripeness > 80.2” and “apples with ripeness ≤ 80.2 and sugar content between 12.6 and 13.5” exhibited a high level of aroma intensity in this study (Figure 8). It is unclear whether the variation is due to measurement time or differences in environment in which apples were grown; however, the aroma sensor values can be used to identify apple characteristics that differ in sugar content and ripeness.

Based on the results presented above, the e-nose device used in this study demonstrated a correlation with the amount of VOCs in apples. Evaluation of the measurement method using fragrances showed both high repeatability and intermediate precision, with less than 10% variation. Furthermore, the results indicated a certain level of stability, even on different measurement days.

Furthermore, no significant correlation was found between the e-nose sensor values on the calyx side of the apples and the sugar content and ripeness measured by the optical sensors. This suggests that the e-nose sensor values can be used as an index to determine the features of apples that are distinct from their sugar content and ripeness. Because the aroma of apples is known to affect palatability [21], it is expected that e-nose systems can be used to add value to the aroma.

This research had several limitations which should be addressed in future studies. First, the effect of ethylene on the sensor value was not considered, though the bottom side of apples was known to produce ethylene [22], and the relationship between ethylene and VOC production was reported [23]. Therefore, the effect of ethylene on the sensor value should be investigated. Second, the relationships among apple quality or palatability, aroma intensity, sugar content, and ripeness were not revealed in this study; a comparison of the sensory evaluation scores may be helpful in elucidating these relationships. 

## 4. Conclusions

Aroma measurements of apple samples were conducted from the calyx side using an e-nose sensor device and direct mass spectrometry. The e-nose sensor values tended to increase in accordance with the intensities of the signals detected by direct mass spectrometry. The accuracy of the measurement method was evaluated using an apple lip balm, and the repeatability and intermediate precision were within 1.36–9.96%. Additionally, no correlation was found between the values of the e-nose sensor and that of the sugar content or ripeness. Thus, measuring apple aroma from the calyx side can be considered as an index for identifying its features. 

## 5. Patents

The author is the inventor of a pending patent in Japan related to the e-nose system used in this study (Application No. 2020-192259).

## Figures and Tables

**Table 1:** Detected molecules and their intensity from apple calyx side aromas.

Molecules	MW	Low		Medium		High		*p*-Value
		Average	SD	Average	SD	Average	SD	
C_4_H_8_O_2_	89.0597	446,425.0	41,647.57	858,083.0 ^#^	101,785.00	901,960.3 ^#^	60,553.19	0.554
C_5_H_10_O_2_	103.0754	2,142,152.0	374,563.86	3,613,496.3	652,776.12	5,090,150.3 ^#^	771,583.74	0.003 *
C_6_H_12_O_2_	117.091	4,082,257.0	427,804.85	6,282,441.3 ^#^	475,569.82	5,623,528.3 ^#^	239,404.42	0.001 *
C_7_H_14_O_2_	131.1067	213,769.7	21,742.91	290,570.0 ^#^	24,511.42	299,218.7 ^#^	14,073.09	0.004 *
C_8_H_16_O_2_	145.1223	1,787,431.0	168,114.28	2,489,897.3 ^#^	215,742.40	2,777,455.0 ^#^	187,333.04	0.002 *
C_9_H_18_O_2_	159.138	166,709.0	8951.05	469,390.0 ^#^	61,167.42	616,414.3 ^#^	89,630.53	0.0003 *
C_10_H_20_O_2_	173.1536	716,778.3	128,842.77	3,041,954.7 ^#^	577,358.99	3,015,308.0 ^#^	613,751.16	0.002 *
C_11_H_22_O_2_	187.1693	1,967,774.0	547,087.39	4,809,816.3 ^#^	1,011,165.44	6,857,352.7 ^#^	1,288,562.06	0.003 *
C_12_H_24_O_2_	201.1849	2,528,008.0	570,220.21	5,359,085.3 ^#^	802,980.68	5,358,540.0 ^#^	459,690.10	0.002 *
Total	—	13,604,879.0	2,133,274.22	26,356,651.3	3,459,975.26	29,637,967.3	3,087,390.22	0.001 *

* Significant difference according to one-way ANOVA. ^#^ Significant difference with low in Tukey’s HSD Test (*p* < 0.05).

**Table 2:** Repeatability and intermediate precision of lip balm measurements.

Area of Apple Lip Balm (cm^2^)	Sensor Values (Mean)	Repeatability (RSD%)	Intermediate Precision (RSD%)
0	146.2	1.36	9.96
0.25	208.2	4.98	6.23
1	260.1	7.81	8.21
4	363.7	6.25	7.88

**Table 3:** Measurement of the 20 apple samples’ aroma using e-nose, optical sugar meter, and optical ripeness meters.

Apples	E-Nose Values (Mean)	Standard Deviation	RSD%	Sugar Content (%)	Ripeness (%)
A	240.3	6.03	2.51	12.4	59.7
B	317.3	11.72	3.69	12.8	76.3
C	308.0	11.27	3.66	13.0	63.7
D	290.0	11.00	3.79	13.7	85.0
E	258.0	9.54	3.70	11.5	73.3
F	272.3	7.37	2.71	12.4	78.0
G	269.0	5.29	1.97	11.3	66.7
H	258.3	5.69	2.20	15.5	76.3
I	223.3	3.51	1.57	11.4	74.7
J	336.7	32.08	9.53	14.7	80.7
K	320.7	10.69	3.33	14.5	85.3
L	234.3	16.26	6.94	14.7	63.3
M	289.3	7.09	2.45	13.4	55.3
N	213.0	4.00	1.88	14.5	68.7
O	223.7	10.02	4.48	14.0	70.3
P	207.7	9.07	4.37	14.5	77.7
Q	253.0	2.00	0.79	12.1	79.7
R	237.7	4.51	1.90	13.6	76.7
S	285.0	19.52	6.85	12.4	36.3
T	218.3	12.42	5.69	13.6	69.7
Mean	262.8	9.95	3.70	13.3	70.9

## Data Availability

The data supporting the findings of this study are available from the corresponding author upon request with the permission of NARO.
